# GTB-PPI: Predict Protein–protein Interactions Based on L1-regularized Logistic Regression and Gradient Tree Boosting

**DOI:** 10.1016/j.gpb.2021.01.001

**Published:** 2021-01-27

**Authors:** Bin Yu, Cheng Chen, Hongyan Zhou, Bingqiang Liu, Qin Ma

**Affiliations:** 1 School of Life Sciences, University of Science and Technology of China, Hefei 230027, China; 2 College of Mathematics and Physics, Qingdao University of Science and Technology, Qingdao 266061, China; 3 Artificial Intelligence and Biomedical Big Data Research Center, Qingdao University of Science and Technology, Qingdao 266061, China; 4 School of Mathematics, Shandong University, Jinan 250100, China; 5 Department of Biomedical Informatics, College of Medicine, The Ohio State University, Columbus, OH 43210, USA

**Keywords:** Protein–protein interaction, Feature fusion, L1-regularized logistic regression, Gradient tree boosting, Machine learning

## Abstract

**Protein–protein interactions** (PPIs) are of great importance to understand genetic mechanisms, delineate disease pathogenesis, and guide drug design. With the increase of PPI data and development of **machine learning** technologies, prediction and identification of PPIs have become a research hotspot in proteomics. In this study, we propose a new prediction pipeline for PPIs based on **gradient tree boosting** (GTB). First, the initial feature vector is extracted by fusing pseudo amino acid composition (PseAAC), pseudo position-specific scoring matrix (PsePSSM), reduced sequence and index-vectors (RSIV), and autocorrelation descriptor (AD). Second, to remove redundancy and noise, we employ **L1-regularized logistic regression** (L1-RLR) to select an optimal feature subset. Finally, GTB-PPI model is constructed. Five-fold cross-validation showed that GTB-PPI achieved the accuracies of 95.15% and 90.47% on *Saccharomyces cerevisiae* and *Helicobacter pylori* datasets, respectively. In addition, GTB-PPI could be applied to predict the independent test datasets for *Caenorhabditis elegans*, *Escherichia coli*, *Homo sapiens*, and *Mus musculus*, the one-core PPI network for CD9, and the crossover PPI network for the Wnt-related signaling pathways. The results show that GTB-PPI can significantly improve accuracy of PPI prediction. The code and datasets of GTB-PPI can be downloaded from https://github.com/QUST-AIBBDRC/GTB-PPI/.

## Introduction

Knowledge of protein–protein interactions (PPIs) can help to probe the mechanisms underlying various biological processes, such as DNA replication, protein modification, and signal transduction [1], [2]. The accurate understanding and analysis of PPIs can reveal multiple functions at the molecular and proteome levels, which has become a research hotspot [3], [4]. However, web-lab identification methods suffer from incomplete and false prediction problems [5]. Alternatively, employing reliable bioinformatics methods for PPI prediction could provide candidates for subsequent experimental validation in a cost-effective way.

Compared with structure-based methods, sequence-based methods are straightforward and do not require *a priori* information, which have been widely used. Martin et al. [6] proposed the signature kernel method to extract protein sequence feature information, but they did not use physicochemical property information. Subsequently, Guo et al. [7] employed seven physicochemical properties of amino acids to predict PPIs by combining autocovariance and support vector machine (SVM).

Different feature extraction methods can complement each other, and prediction accuracy can be improved by effective feature fusion [8], [9]. For instance, Du et al. [8] constructed a PPI prediction framework called DeepPPI, which employed deep neural networks as the classifier. They fused amino acid composition information-based features and physiochemical property-based sequence features. However, presence of information redundancy, noise, and excessively high dimensionalities after feature fusion would affect the classification accuracy. You et al. [10] used the minimum redundancy maximum relevance (mRMR) to determine important and distinguishable features to predict PPIs based on SVM.

Ensemble learning systems can achieve higher prediction performance than a single classifier. To our knowledge, Jia et al. [11] combined seven random forest (RF) classifiers according to voting principles. As an ensemble learning method, gradient tree boosting (GTB) has been widely applied in miRNA–disease association [12], drug–target interaction [13], and RNA-binding residue prediction [14]. GTB outperforms SVM and RF, showing superior model generalization performance.

Although a large number of algorithms have been proposed and developed, challenges remain for sequenced-based PPI predictors currently available. First, the sequence-only-based information of PPIs is not fully represented and elucidated, and satisfactory results cannot be obtained by merely adjusting individual parameters. Multi-information fusion is a very useful strategy through fusing multiple descriptors, such as pseudo amino acid composition (PseAAC) and pseudo position-specific scoring matrix (PsePSSM), which have been widely applied in PPI prediction [15], Gram-negative protein localization prediction [16], identification of submitochondrial locations [17], and apoptosis protein localization prediction [18]. Secondly, there is a severe data imbalance problem in PPI prediction. The number of non-interacting protein pairs is much higher than that of interacting protein pairs. Currently, machine learning methods cannot deal with such problems well and could result in poor overall performance when dealing with imbalanced data [19].

To overcome the aforementioned limitation of machine learning methods, this study proposes a new PPI prediction pipeline called GTB-PPI. First, we fuse PseAAC, PsePSSM, reduced sequence and index-vectors (RSIV), and autocorrelation descriptor (AD) to extract amino acid composition-based information, evolutionary information, and physicochemical information. To retrieve effective details representing PPIs without losing important and reliable characteristic information, L1-regularized logistic regression (L1-RLR) is first utilized for PPI prediction to eliminate redundant features. At the same time, we employ GTB as a classifier to bridge the gap between the extracted PPI features and class label. Our data show that the PPI prediction performance of GTB is better than that of SVM, RF, Naïve Bayes (NB), and *K* nearest neighbors (KNN) classifiers. The linear combination of decision trees can fit the PPI data well. When applied to the network prediction, GTB-PPI obtains the accuracy values of 93.75% and 95.83% for the one-core PPI network for CD9 and the crossover PPI network for the Wnt-related signaling pathways, respectively.

## Method

### Data source

The *Saccharomyces cerevisiae* PPI dataset was obtained from the Database of Interacting Proteins (DIP) (DIP: 20070219) [7]. Protein sequences consisting of < 50 amino acid residues or showing sequence identity ≥ 40% via CD-HIT [20] were removed. Thus, 5594 interacting protein pairs are considered as positive samples; 5594 protein pairs with different subcellular location information are selected as negative samples, and their location information is obtained from Swiss-Prot. The *Helicobacter pylori* PPI dataset was constructed before [6], which contains 2916 samples (1458 PPI pairs and 1458 non-PPI pairs).

Four independent PPI datasets [21] were also used to test the performance of GTB-PPI. These datasets are obtained from *Caenorhabditis elegans* (4013 interacting pairs), *Escherichia coli* (6954 interacting pairs), *Homo sapiens* (1412 interacting pairs), and *Mus musculus* (313 interacting pairs). The number of unique proteins in each dataset is shown in Table S1.

### Feature extraction

We fuse PseAAC, PsePSSM, RSIV, and AD to extract the PPI feature information, including sequence-based features, evolutionary information features, and physicochemical property features. The detailed descriptions of methods are presented in File S1.

### L1-RLR

L1-RLR is an embedded feature selection method. Given the sample dataset $D=\{\left(x_{1},y_{1}\right),\left(x_{2},y_{2}\right),\cdot \cdot \cdot ,\left(x_{m},y_{m}\right)\}$, L1-RLR can be transformed into an unconstrained optimization problem.

(1) $$\min_{w}f\left(\omega \right)={∥\omega ∥}_{1}+C\left(\sum_{i=1}^{l}\log(1+e^{-\omega^{T}x_{i}})+\sum_{i:y_{i}=-1}\omega^{T}x_{i}\right)$$

where ${∥\cdot ∥}_{1}$ represents the L1 norm; l is the number of samples; ω represents the weight coefficient; and C represents penalty term, which determines the number of selected features. We use the coordinate descent algorithm in LIBLINEAR [22] to solve Equation (1).

### GTB

GTB can be used to aggregate multiple decision trees [23], [24], [38], [40], [42]. Different from other ensemble learning algorithms, GTB fits residual of the regression tree at each iteration using negative gradient values of loss.

GTB can be expressed as the relationship between the label y and the vector of input variables x, which are connected via a joint probability distribution p(x,y). The goal of GTB is to obtain the estimated function $\hat{F}\left(x\right)$ through minimizing L(y,F(x)):

(2) $$\hat{F}=\arg\;\min_{F}E_{x,y}\left[L\left(y,F\left(x\right)\right)\right]$$

Let $h_{m}\left(x\right)$ be the m-th decision tree and $J_{m}$ indicates number of its leaves. The tree partitions the input space into $J_{m}$ disjoint regions $R_{1,m},R_{2,m},\cdot \cdot \cdot ,R_{J_{m},m}$ and predicts a numerical value $b_{\mathrm{jm}}$ for each region $R_{\mathrm{jm}}$. The output of $h_{m}\left(x\right)$ can be described as:

$$h_{m}\left(x\right)=\sum_{j=1}^{J_{m}}b_{\mathrm{jm}}1_{R_{\mathrm{jm}}}\left(x\right)$$

Then the value of $\gamma_{m}$ can be obtained using steepest descent to fulfill the GTB model:

(3) $$\gamma_{m}=\arg\;\min_{\gamma }\sum_{i=1}^{n}L(y_{i},F_{m-1}\left(x_{i}\right)+\gamma h_{m}\left(x_{i}\right))$$

where $F_{m-1}\left(x\right)$ represents an estimated function.

The iterative criterion of GTB is shown using Equation (4).

(4) $$F_{m}\left(x\right)=F_{m-1}\left(x\right)+\gamma_{m}h_{m}\left(x\right)$$

where iterations are set as M, and GTB model is $\hat{F}\left(x\right)=F_{M}\left(x\right)$.

GTB can complement the weak learning ability of decision tree, thus improving the ability of representation, optimization, and generalization. GTB can capture higher-order information and is invariant to scaling of sample data. GTB can effectively avoid overfitting condition by weighting combination scheme. GTB-PPI uses the GTB algorithm of Scikit-learn [25].

### Performance evaluation

In GTB-PPI pipeline, recall, precision, overall prediction accuracy (ACC), and Matthews correlation coefficient (MCC) are used to evaluate the model performance [8]. The definitions are as follows:

(5) $$\mathrm{Recall}=\frac{\mathrm{TP}\;}{\mathrm{TP}\;+\;FN}$$

(6) $$\mathrm{Precision}=\frac{\mathrm{TP}}{\mathrm{TP}\;+\;FP}\;$$

(7) $$\mathrm{ACC}=\frac{\mathrm{TP}\;+TN}{\mathrm{TP}\;+TN\;+\;FN\;+\;FP}\;$$

(8) $$\mathrm{MCC}=\frac{\mathrm{TP}\times TN-FP\times FN}{\sqrt{\left(TP+FP)(TN+FN)(TP+FN)(TN+FP\right)}}$$

TP indicates the number of predicted PPI samples found in PPI dataset; TN indicates the number of non-PPI samples correctly predicted; FP and FN indicate false positive and false negative, respectively. Receiver operating characteristic (ROC) curve [26], precision–recall (PR) curve [27], area under ROC curve (AUROC), and area under PR curve (AUPRC) are also used to evaluate the generalization ability of GTB-PPI.

## Results and discussion

### GTB-PPI pipeline

**Figure 1:**
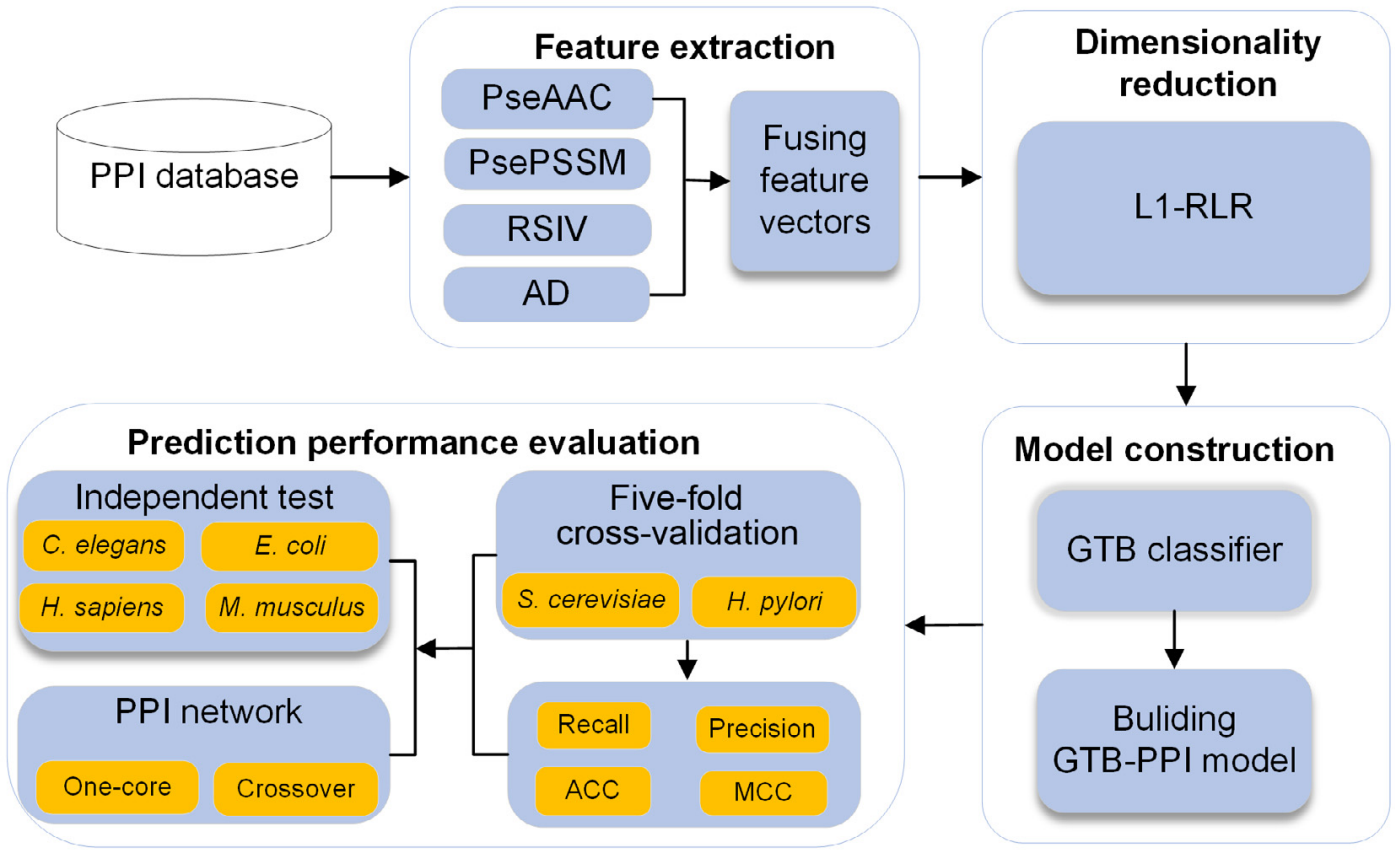
**Overall framework of GTB-PPI for PPI prediction** First, the benchmark datasets are collected. Second, PseAAC, PsePSSM, RSIV, and AD are used for feature extraction. Third, the L1-RLR is employed for dimensionality reduction. Fourth, we use GTB to predict PPIs and GTB-PPI model is constructed. Finally, five-fold cross-validation, independent test, and PPI network are employed to evaluate GTB-PPI. PseAAC, pseudo amino acid composition; PsePSSM, pseudo-position-specific scoring matrix; RSIV, reduced sequence and index-vectors; AD, autocorrelation descriptor; GTB, gradient tree boosting; PPI, protein–protein interaction; ACC, overall prediction accuracy; MCC, Matthews correlation coefficient; L1-RLR, L1-regularized logistic regression.

The pipeline of GTB-PPI for predicting PPIs is shown in Figure 1, which can be implemented using MATLAB 2014a and Python 3.6. There are five steps of GTB-PPI as described below.

#### *Data input*

The input values of GTB-PPI are PPI samples, non-PPI samples, and the corresponding binary labels.

#### *Feature extraction*

PseAAC, PsePSSM, RSIV, and AD are fused to transform the protein character signal into numerical signal. 1) Amino acid sequence composition and sequence order information are obtained using PseAAC to construct the 20+λ dimensional vectors. 2) PSSM matrix of the protein sequence is obtained and 20+20×ξ features are extracted based on PsePSSM. 3) Feature information is extracted using RSIV according to the six physicochemical properties. Each protein sequence is constructed as 120 + 77 = 197 dimensional vectors. 4) Protein sequence is transformed into 3×7×lag dimensional vectors by Morean-Broto autocorrelation (MBA), Moran autocorrelation (MA), and Geary autocorrelation (GA). λ, ξ, and lag are the hyperparameters of GTB-PPI, and their detailed meaning can be seen in File S1.

#### *Dimensionality reduction*

L1-RLR is first employed to remove redundant features by adjusting the penalty parameters in logistic regression. The performance of L1-RLR is then compared with that of semi-supervised dimension reduction (SSDR), principal component analysis (PCA), kernel principal component analysis (KPCA), factor analysis (FA), mRMR, and conditional mutual information maximization (CMIM) on *S. cerevisiae* and *H. pylori* datasets.

#### *PPI prediction based on GTB*

According to step 2 for feature extraction and step 3 for dimensionality reduction, L1-RLR is used to better capture the sequence representation details. In this way, GTB-PPI model can be constructed using GTB as the classifier.

#### *PPI prediction on independent test datasets and network datasets*

The optimal feature set representing PPIs can be obtained through feature encoding, fusion, and selection. GTB is employed to predict the binary labels on four independent test datasets and two network datasets.

### Parameter optimization of PseAAC, PsePSSM, and AD

It is essential to optimize parameters of PseAAC, PsePSSM, and AD for GTB-PPI predictor construction. We implement the hyperparameter optimization through five-fold cross-validation.

**Figure 2:**
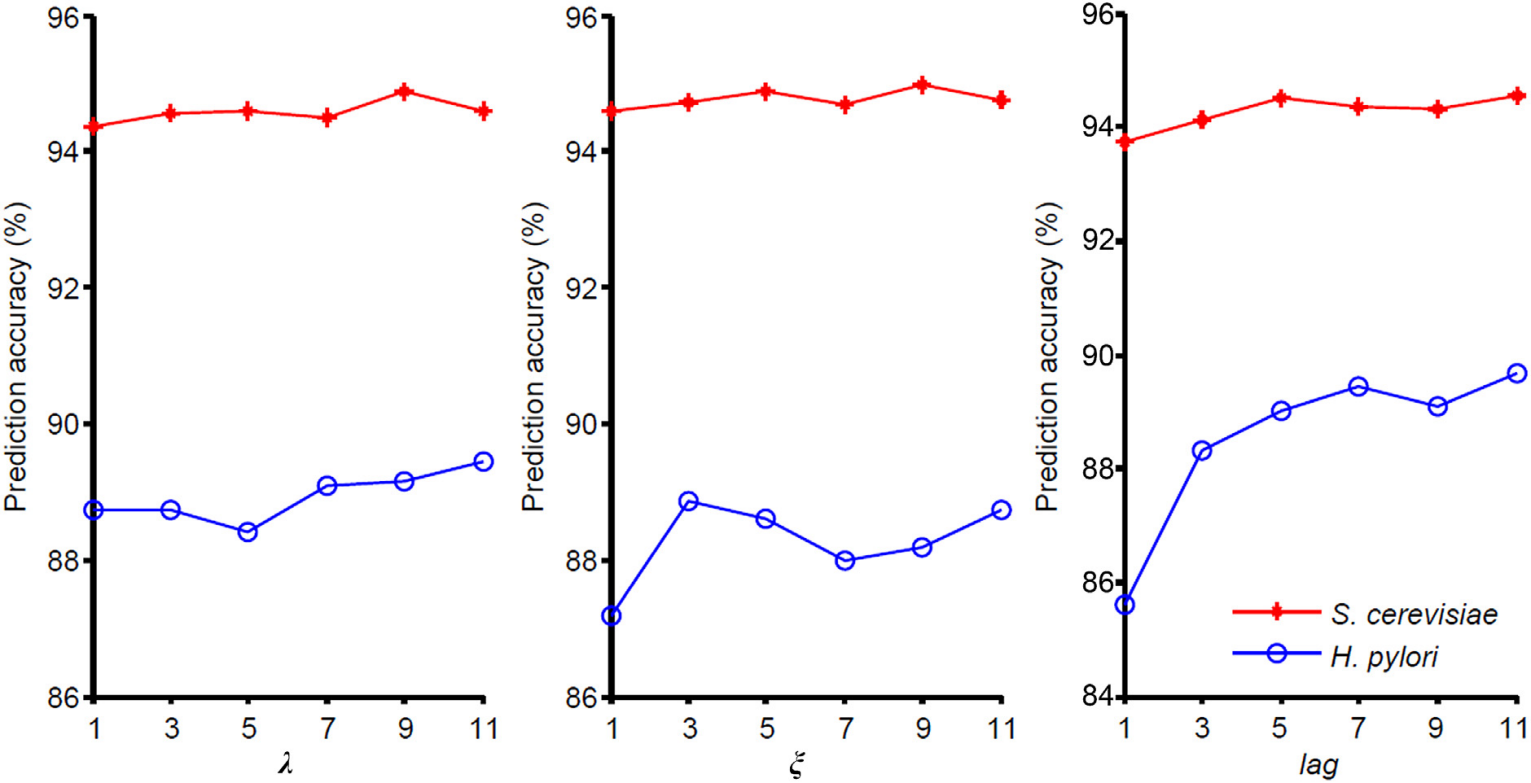
**Prediction results of different parameters**λ**,**ξ**, and**lag**on the *S. cerevisiae* and *H. pylori*datasets** The λ, ξ, and lag are the parameters that need to be adjusted in PseAAC, PsePSSM, and AD, respectively.

To extract features from the sequence, the values for λ of PseAAC, ξ of PsePSSM, and lag of AD should be determined. We set the values of λ as 1, 3, 5, 7, 9, and 11; similarly, values for ξ and lag are also set as 1, 3, 5, 7, 9, and 11 in order. GTB is then used to predict the binary labels (Tables S2–S4). As shown in Figure 2, the prediction performance on *S. cerevisiae* and *H. pylori* datasets changed with the alteration in the values of the respective parameters. For the parameter λ in PseAAC, the highest prediction performance for these two datasets was obtained at different λ values: the optimal λ value for *S. cerevisiae* is 9, while the optimal λ value of *H. pylori* is 11. Considering that PseAAC generates fewer dimensional vectors than the other three feature extraction methods (PsePSSM, RSIV, and AD), we choose the optimal parameter λ=11 to mine more PseAAC information. The parameter selection of ξ and lag can be found in File S2. In summary, for each protein sequence, PseAAC extracts 20+11=31 features, PsePSSM obtains 20+20×9=200 features, the dimension of RSIV is 197, and AD encodes 3×7×11=231 features. We can obtain 659-dimensional vectors by fusing all four coding methods. Then the 1318-dimensional feature vectors are constructed by concatenating two sequences of protein pairs.

### Effect of dimensionality reduction

**Figure 3:**
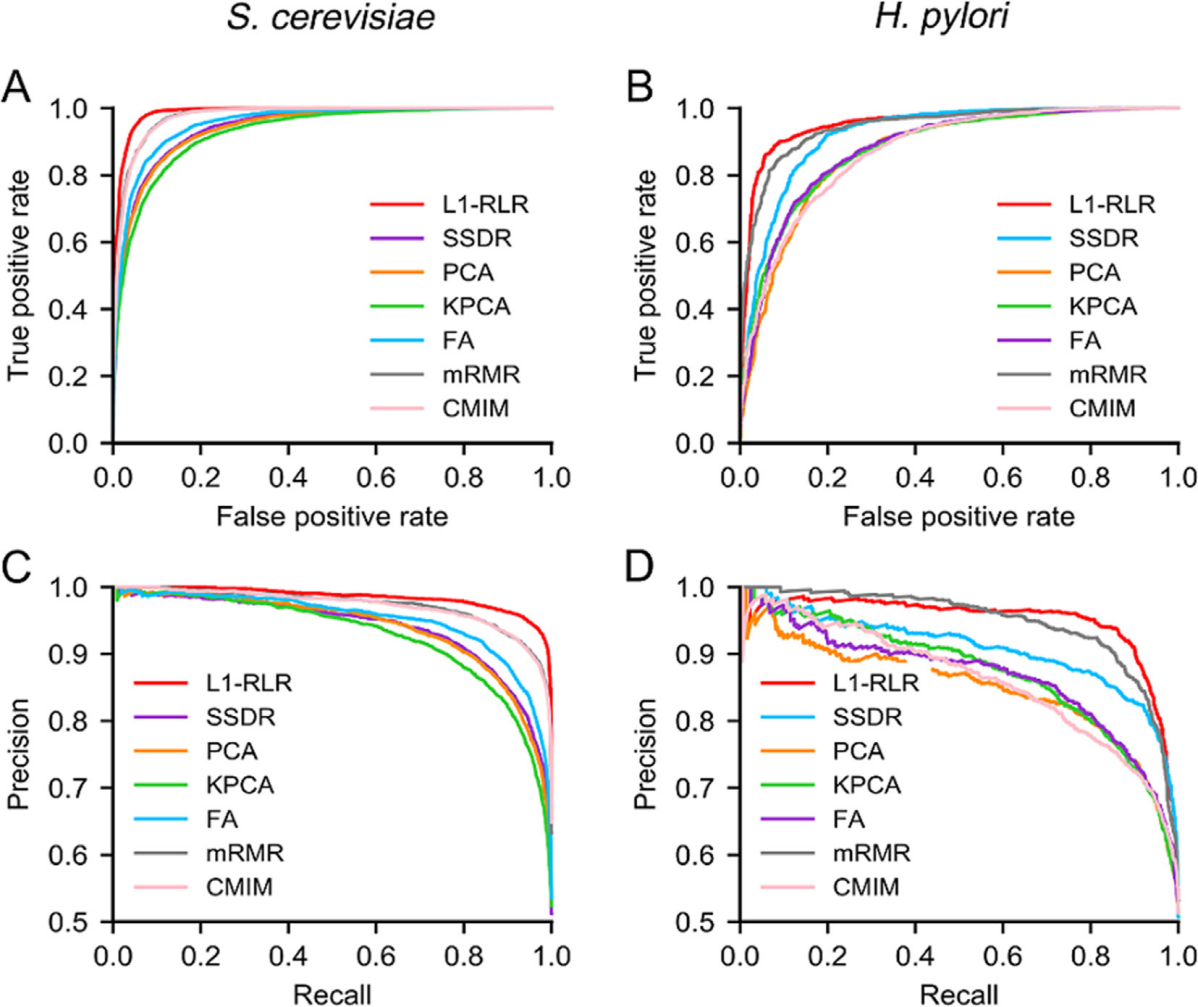
**Prediction performance of different dimensionality reduction methods** **A.** ROC curves of L1-RLR, SSDR, PCA, KPCA, FA, mRMR, and CMIM for the *S. cerevisiae* dataset. **B.** ROC curves of L1-RLR, SSDR, PCA, KPCA, FA, mRMR, and CMIM for the *H. pylori* dataset. **C.** PR curves of L1-RLR, SSDR, PCA, KPCA, FA, mRMR, and CMIM for the *S. cerevisiae* dataset. **D.** PR curves of L1-RLR, SSDR, PCA, KPCA, FA, mRMR, and CMIM for the *H. pylori* dataset. ROC, receiver operating characteristic; SSDR, semi-supervised dimension reduction; PCA, principal component analysis; KPCA, kernel principal component analysis; FA, factor analysis; mRMR, minimum redundancy maximum relevance; CMIM, conditional mutual information maximization; PR, precision–recall.

**Supplementary figure S1:**
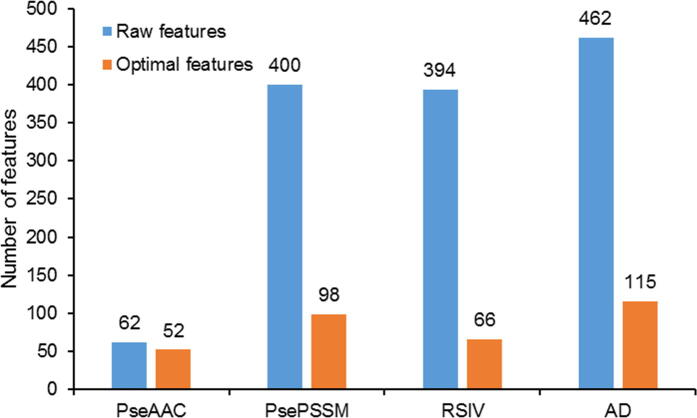
**Number of feature subsets with different feature extraction methods on the *S. cerevisiae* dataset**. Raw features represent the initial feature dimensions and optimal features represent the selected features.

**Supplementary figure S2:**
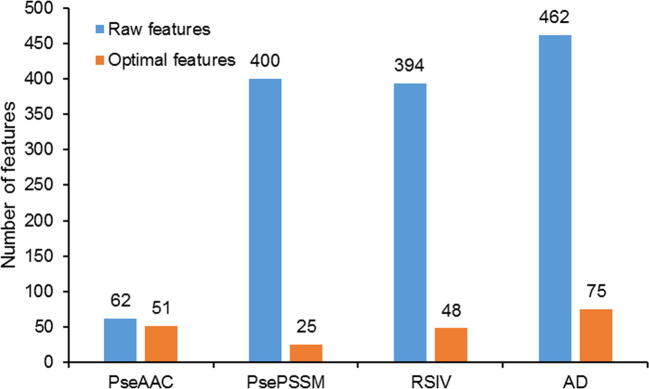
**Number of feature subsets with different feature extraction methods on the *H. pylori* dataset.** Raw features represent the initial feature dimensions and optimal features represent the selected features.

L1-RLR can effectively improve prediction performance with higher computational efficiency. The process of parameter selection is described in File S3. To evaluate the performance of L1-RLR (C=1), we compared its prediction performance with SSDR [28], PCA [29] (setting of contribution rate is shown in Table S5), KPCA [30] (adjustment of contribution rate is shown in Table S6), FA [31], mRMR [32], and CMIM [33] (Table S7). ROC and PR curves of different dimensionality reduction methods are shown in Figure 3. The AUROC and AUPRC are shown in Table S8. The numbers of raw features and optimal features can be obtained in Figures S1 and S2.

As shown in Figure 3A and B, ROC curves for both the *S. cerevisiae* and *H. pylori* datasets show that the L1-RLR has superior model performance. For the *S. cerevisiae* dataset, the AUROC value of L1-RLR is 0.9875, which is 4.55%, 4.83%, 6.13%, 3.21%, 1.07%, and 1.09% higher than that of SSDR, PCA, KPCA, FA, mRMR, and CMIM, respectively (Table S8). For the *H. pylori* dataset, the AUROC value of L1-RLR is 0.9559, which is 3.47%, 9.80%, 8.59%, 8.33%, 1.04%, and 9.55% higher than that of SSDR, PCA, KPCA, FA, mRMR, and CMIM, respectively (Table S8). As shown in Figure 3C and D, in PR curves, L1-RLR almost obtains the highest precision value at corresponding recall value. The AUPRC values of L1-RLR are 1.22%–6.21% and 0.36%–11.94% higher than the other six dimensionality reduction methods on the *S. cerevisiae* and *H. pylori* datasets, respectively (Table S8). These results indicate that L1-RLR can effectively remove the redundant features without losing important information. The effective features related to PPIs could be fed into a GTB classifier, generating a reliable GTB-PPI prediction model.

### Selection of classifier algorithms

**Figure 4:**
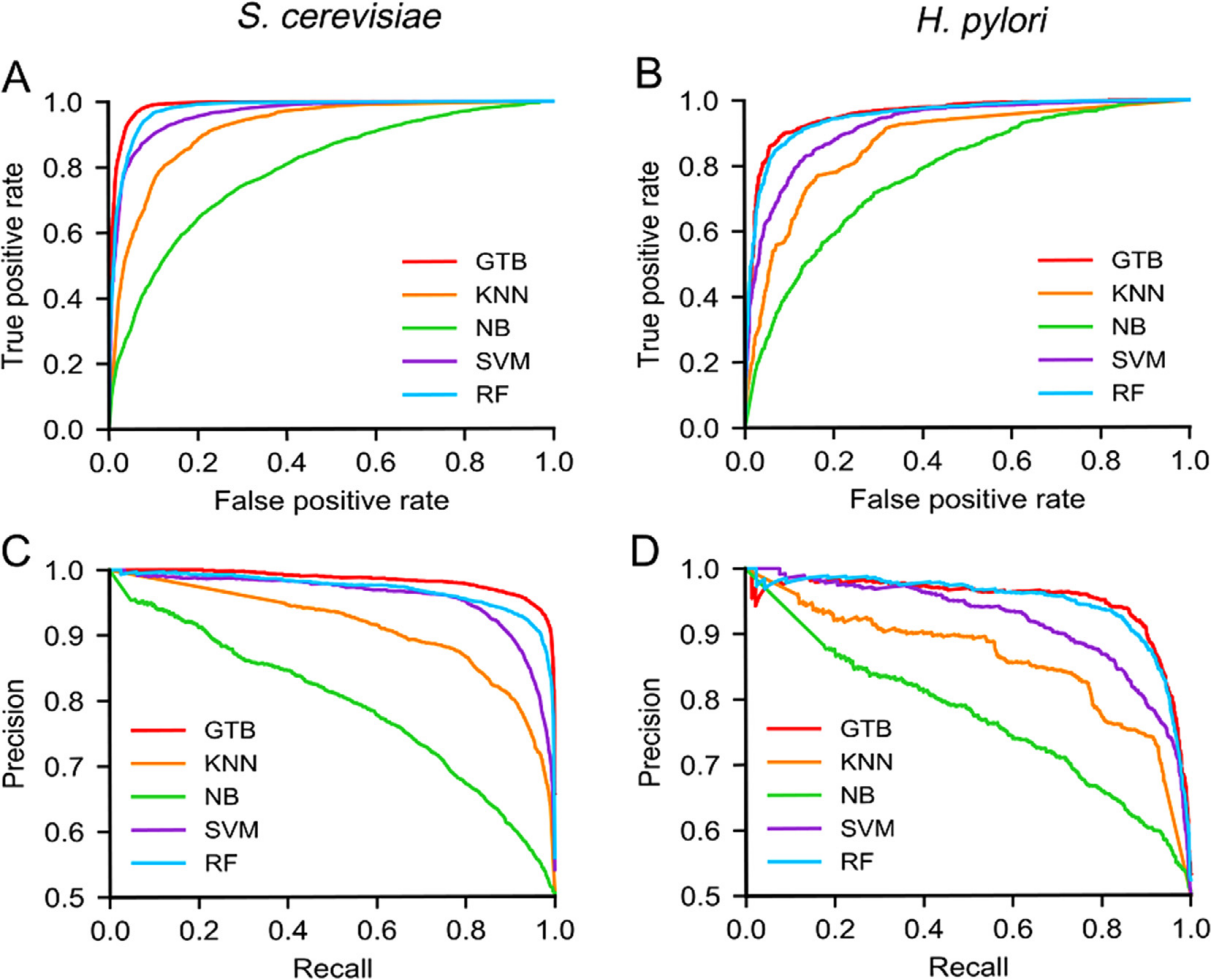
**Comparison of GTB with KNN, NB, SVM, and RF classifiers** **A.** ROC curves of KNN, NB, SVM, RF, and GTB for the *S. cerevisiae* dataset. **B.** ROC curves of KNN, NB, SVM, RF, and GTB for the *H. pylori* dataset. **C.** PR curves of KNN, NB, SVM, RF, and GTB for the *S. cerevisiae* dataset. **D.** PR curves of KNN, NB, SVM, RF, and GTB for the *H. pylori* dataset. KNN, *K* nearest neighbors; NB, Naïve Bayes; SVM, support vector machine; RF, random forest.

**Supplementary figure S3:**
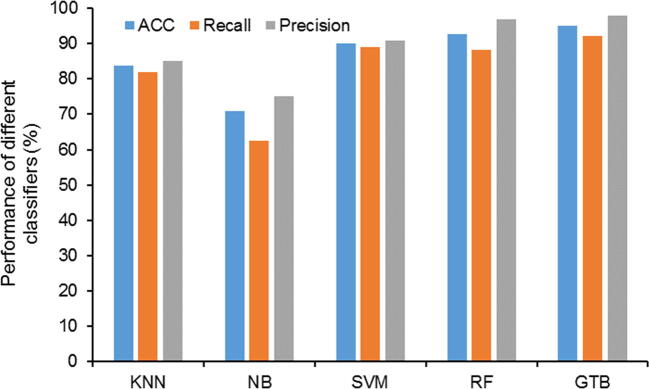
**Comparison of prediction results with different classifiers on the *S. cerevisiae* dataset**.

**Supplementary figure S4:**
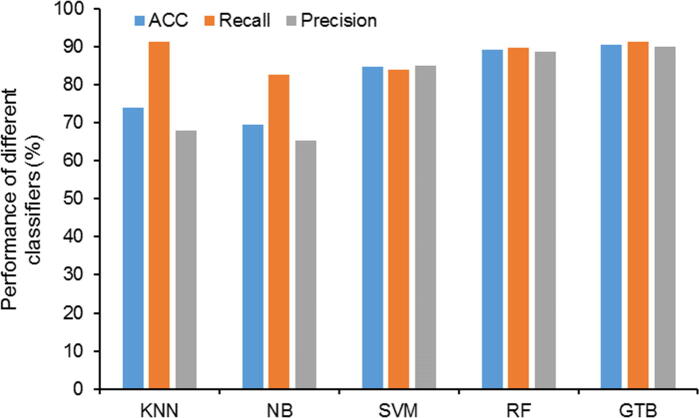
**Comparison of prediction results with different classifiers on the *H. pylori* dataset**.

GTB is used as a classifier with the number of iterations set to 1000 and loss function set as “deviance”. The prediction results of other four classifiers are also provided via five-fold cross-validation, including KNN [34] (number of neighbors = 3) (Table S9), NB [35], SVM [36] (recursive feature elimination as the kernel function), and RF [37] (number of the base decision trees = 1000) (Table S10). The prediction results of KNN, SVM, NB, RF, and GTB on the *S. cerevisiae* and *H. pylori* datasets are shown in Table S11 and Figures S3 and S4. We also obtain the ROC and PR curves (Figure 4) and AUROC and AUPRC values for different classifiers (Table S12).

As shown in Figure 4A and B, ROC curves for both the *S. cerevisiae* and *H. pylori* datasets show that the GTB classifier outperforms than KNN, NB, SVM, and RF. The AUROC values of GTB are 1.16%–24.65% and 0.53%–22.95% higher than the other four classifier methods on the *S. cerevisiae* and *H. pylori* datasets, respectively (Table S12). As shown in Figure 4C and D, the prediction performance of GTB is superior to KNN, NB, SVM, and RF. The AUPRC values of GTB are 1.42%–24.32% and 0.22%–24.56% higher than the other four classifier methods on the *S. cerevisiae* and *H. pylori* datasets, respectively (Table S12). These results demonstrate that GTB-PPI can accurately indicate whether a pair of proteins interact with each other within the *S. cerevisiae* or *H. polyri* dataset. GTB is an ensemble method using boosting algorithm that can achieve superior generalization performance over a single learner. Specially, RF achieves worse performance than GTB, because all the base decision trees of RF are treated equally. If the base classifier’s prediction performance is biased, the final ensemble classifier may get the unreliable and biased predicted results. GTB can utilize steepest descent step algorithm to bridge the gap between the sequence and PPI label information.

### Comparison of GTB-PPI with other PPI prediction methods

To verify the validity of the GTB-PPI model, we compare GTB-PPI with ACC+SVM [7], DeepPPI [8], and other state-of-the-art methods on the *S. cerevisiae* and *H. pylori* datasets.

As shown in Table 1, for the *S. cerevisiae* dataset, compared with other existing methods, the ACC of GTB-PPI increases by 0.14%–9.00%; the recall of GTB-PPI is 0.15% higher than DeepPPI [8] and 1.54% higher than MCD+SVM [10]; the precision of GTB-PPI is 1.32% higher than DeepPPI [8] and 0.81% higher than MIMI+NMBAC+RF [41].

**Table 1:** **Performance comparison of GTB-PPI with other state-of-the-art predictors on the *S. cerevisiae* dataset**

*Note*: N/A means not available. Data are presented as mean ± SD. ACC, overall prediction accuracy; MCC, Matthews correlation coefficient; SVM, support vector machine; Code4, feature concatenation; KNN, *K* nearest neighbors; MCD, multi-scale continuous and discontinuous feature extraction; MLD, multi-scale local feature representation; RF, random forest; MIMI, multivariate mutual information; NMBAC, normalized Morean-Broto autocorrelation; LRA, low-rank approximation; DeepPPI, deep neural network for protein–protein interaction prediction; GTB-PPI, gradient tree boosting for protein–protein interaction prediction. Holdout validation is adopted in the previous report [7].

As shown in Table 2, for the *H. pylori* dataset, the performance of GTB-PPI is better than other tested predictors. In terms of ACC, GTB-PPI is 2.88%–7.07% higher than other methods (7.07% higher than SVM [6], 4.24% higher than DeepPPI [8], and 3.73% higher than DCT+WSRC [45]). At the same time, the recall of GTB-PPI is 1.71%–12.15% higher than other methods (4.72% higher than DCT+WSRC [45] and 7.91% higher than MCD+SVM [10]). The precision of GTB-PPI is 1.76%–5.67% higher than other methods (4.29% higher than SVM [6] and 5.67% higher than DeepPPI [8]).

**Table 2:** **Performance comparison of GTB-PPI with other state-of-the-art predictors on the *H. pylori* dataset** ([6], [43], [44], [45], [10], [41], [8].)

*Note*: N/A means not available. Data are presented as mean ± SD. SVM, WSR, and Ensemble of HKNN use ten-fold cross-validation. WSR, weighted sum rule; DCT, discrete cosine transform; WSRC, weighted sparse representation classifier.

### PPI prediction on independent test datasets

The performance of GTB-PPI can also be evaluated using cross-species datasets. After the feature extraction, fusion, and selection, the *S. cerevisiae* dataset is used as a training set to predict PPIs of four independent test datasets.

As shown in Table 3, for the *C. elegans* dataset, the ACC of GTB-PPI is 0.26% higher than MIMI+NMBAC+RF [41], 4.71% higher than MLD+RF [39], and 11.23% higher than DCT+WSRC [45], but 2.42% lower than DeepPPI [8]. For the *E. coli* dataset, the ACC of GTB-PPI (94.06%) is 1.26%–27.98% higher than DeepPPI (92.19%) [8], MIMI+NMBAC+RF (92.80%) [41], MLD+RF (89.30%) [39], and DCT+WSRC (66.08%) [45]. For the *H. sapiens* dataset, the ACC of GTB-PPI (97.38%) is 3.05%–15.16% higher than DeepPPI (93.77%) [8], MIMI+NMBAC+RF (94.33%) [41], MLD+RF (94.19%) [39], and DCT+WSRC (82.22%) [45]. For the *M. musculus* dataset, the ACC of GTB-PPI (98.08%) is 2.23%–18.21% higher than DeepPPI (91.37%) [8], MIMI+NMBAC+RF (95.85%) [41], MLD+RF (91.96%) [39], and DCT+WSRC (79.87%) [45]. The findings indicate that the hypothesis of mapping PPIs from one species to another species is reasonable. We can conclude that PPIs in one organism might have “co-evolve” with other organisms [41].

**Table 3:** **Performance comparison of GTB-PPI with other state-of-the-art predictors on independent datasets**

### PPI network prediction

The graph visualization of the PPI network can provide a broad and informative idea to understand the proteome and analyze the protein functions. We employ GTB-PPI to predict the simple one-core PPI network for CD9 [46] and crossover PPI network for the Wnt-related signaling pathways [47] using the *S. cerevisiae* dataset as a training set.

**Figure 5:**
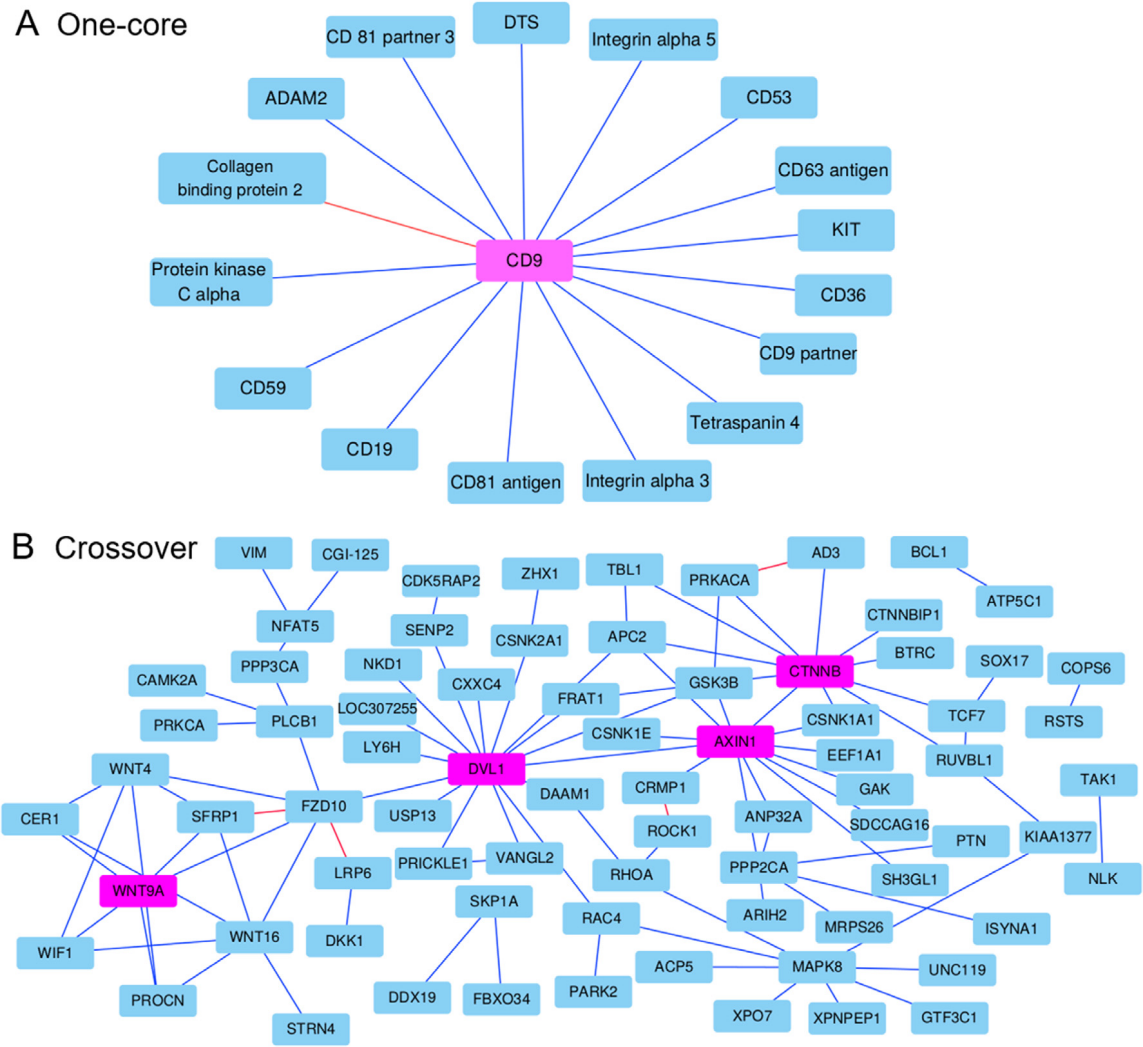
**Prediction results of one-core and crossover networks using GTB-PPI** **A** The prediction performance of one-core network for CD9. CD9 is the core protein, and the others are satellite proteins. 15 of all 16 PPIs are predicted successfully. **B**. The prediction performance of crossover network for the Wnt-related signaling pathways. WNT9A, DVL1, AXIN1, and CTNNB are linked in this work, which are of great importance to the Wnt-related signaling pathways. 92 of the 96 PPI pairs are identified. The blue and red lines represent true and false prediction of PPIs, respectively. The two networks are from Ding et al. [41] and Shen et al. [48].

As shown in Figure 5A, only the interaction between CD9 and Collagen-binding protein 2 is not predicted successfully based on GTB-PPI, which was not predited by Shen et al. [48] either. Compared with Shen et al. [48] and Ding et al. [41], GTB-PPI achieves the superior prediction performance. The ACC is 93.75%, which is 12.50% higher than Shen et al. (81.25%) [48] and 6.25% higher than Ding et al. (87.50%) [41]. As shown in Figure 5B, 92 of the 96 PPI pairs are identified based on GTB-PPI. The ACC is 95.83%, which is 19.79% higher than Shen et al. (76.04%) [48] and 1.04% higher than Ding et al. (94.79%) [41].

The palmitoylation of CD9 could support CD9 to interact with CD53 [49]. In the one-core network for CD9, we can see that the interaction between CD9 and CD53 is predicted successfully based on GTB-PPI. In the crossover PPI network for the Wnt-related signaling pathways, ANP32A, CRMP1, and KIAA1377 are linked to the Wnt signaling pathway via PPIs. The ANP32A has been demonstrated as a potential tumor suppressor [50], and GTB-PPI could predict its interactions with the corresponding proteins. However, the interaction between ROCK1 and CRMP1 is not predicted. It is likely because we use the *S. cerevisiae* dataset as a training set, and ROCK1 and CRMP1 are different organism genes from *S. cerevisiae*. At the same time, ROCK1 is part of the noncanonical Wnt signaling pathway [47], GTB-PPI may not be very effective in this case. A previous study has reported that AXIN1 could interact with multiple proteins [51]. Here, we find that GTB-PPI can predict the interactions between AXIN1 and its satellite proteins, which provides new insights to elucidate the biological mechanism of PPI network.

## Conclusion

The knowledge and analysis of PPIs can help us to reveal the structure and function of protein at the molecular level, including growth, development, metabolism, signal transduction, differentiation, and apoptosis. In this study, a new PPI prediction pipeline called GTB-PPI is presented. First, PseAAC, PsePSSM, RSIV, and AD are concatenated as the initial feature information for predicting PPIs. PseAAC obtains not only the amino acid composition information but also the sequence order information. PsePSSM can mine the evolutionary information and local order information. RSIV can obtain the frequency feature information using the reduced sequence. AD reflects the physicochemical property features on global amino acid sequence. Second, L1-RLR can obtain effective information features related to PPIs without losing accuracy and generalization. Simultaneously, the performance of L1-RLR is superior to SSDR, PCA, KPCA, FA, mRMR, and CMIMs (Figure 3). Finally, the PPIs are predicted based on GTB whose base classifier is a decision tree, which can bridge the gap between amino acid sequence information features and class label. Experimental results show that the PPI prediction performance of GTB is better than that of SVM, RF, NB, and KNN. Especially, in the field of binary PPI prediction, the L1-RLR is used for dimensionality reduction for the first time. The GTB is also first employed as a classifier. In a word, GTB-PPI shows good performance, representation ability, and generalization ability.

## Availability

All datasets and code of GTB-PPI can be obtained on https://github.com/QUST-AIBBDRC/GTB-PPI/.

## CRediT author statement

**Bin Yu:** Conceptualization, Data curation, Formal analysis, Investigation, Methodology, Writing - original draft, Validation, Writing - review & editing. **Cheng Chen:** Data curation, Formal analysis, Investigation, Methodology, Writing - original draft, Validation, Visualization. **Hongyan Zhou:** Formal analysis, Investigation, Methodology, Validation, Visualization. **Bingqiang Liu:** Formal analysis, Investigation, Methodology, Writing - original draft. **Qin Ma:** Data curation, Formal analysis, Investigation, Methodology, Writing - original draft, Writing - review & editing. All authors read and approved the final manuscript.

## Competing interests

The authors declare that they have no known competing financial interests or personal relationships that could have appeared to influence the work reported in this paper.

## Supplementary material

Supplementary File S1 **Feature extraction methods**.

Supplementary File S2 **Parameter selection of *ξ* and *lag***.

Supplementary File S3 **Parameter optimization of L1-RLR**.

## References

[1] Alberts B. (1998). The cell as a collection of protein machines: preparing the next generation of molecular biologists. Cell.

[2] Schadt E.E. (2009). Molecular networks as sensors and drivers of common human diseases. Nature.

[3] Chua H.N., Sung W.K., Wong L. (2006). Exploiting indirect neighbours and topological weight to predict protein function from protein-protein interactions. Bioinformatics.

[4] Sun P.G., Quan Y.N., Miao Q.G., Chi J. (2018). Identifying influential genes in protein-protein interaction networks. Inform Sciences.

[5] Braun P., Tasan M., Dreze M., Barriosrodiles M., Lemmens I., Yu H. (2009). An experimentally derived confidence score for binary protein-protein interactions. Nat Methods.

[6] Martin S., Roe D., Faulon J.L. (2005). Predicting protein-protein interactions using signature products. Bioinformatics.

[7] Guo Y., Yu L., Wen Z., Li M. (2008). Using support vector machine combined with auto covariance to predict protein-protein interactions from protein sequences. Nucleic Acids Res.

[8] Du X.Q., Sun S., Hu C., Yao Y., Yan Y., Zhang Y. (2017). DeepPPI: boosting prediction of protein-protein interactions with deep neural networks. J Chem Inf Model.

[9] Göktepe Y.E., Kodaz H. (2018). Prediction of protein-protein interactions using an effective sequence based combined method. Neurocomputing.

[10] You Z.H., Zhu L., Zheng C.H., Yu H.J., Deng S.P., Ji Z. (2014). Prediction of protein-protein interactions from amino acid sequences using a novel multi-scale continuous and discontinuous feature set. BMC Bioinformatics.

[11] Jia J., Liu Z., Xiao X., Liu B., Chou K.C. (2015). iPPI-Esml: an ensemble classifier for identifying the interactions of proteins by incorporating their physicochemical properties and wavelet transforms into PseAAC. J Theor Biol.

[12] Chen X., Huang L., Xie D., Zhao Q. (2018). EGBMMDA: extreme gradient boosting machine for miRNA-disease association prediction. Cell Death Dis.

[13] He T., Heidemeyer M., Ban F., Cherkasov A., Ester M. (2017). SimBoost: a read-across approach for predicting drug-target binding affinities using gradient boosting machines. J Cheminform.

[14] Tang Y., Liu D., Wang Z., Wen T., Deng L. (2017). A boosting approach for prediction of protein-RNA binding residues. BMC Bioinformatics.

[15] Chen C., Zhang Q., Ma Q., Yu B. (2019). LightGBM-PPI: Predicting protein-protein interactions through LightGBM with multi-information fusion. Chemomet Intell Lab Syst.

[16] Yu B., Li S., Chen C., Xu J.M., Qiu W.Y., Wu X. (2017). Prediction subcellular localization of Gram-negative bacterial proteins by support vector machine using wavelet denoising and Chou’s pseudo amino acid composition. Chemomet Intell Lab Syst.

[17] Yu B., Qiu W.Y., Chen C., Ma A., Jiang J., Zhou H. (2020). SubMito-XGBoost: predicting protein submitochondrial localization by fusing multiple feature information and eXtreme gradient boosting. Bioinformatics.

[18] Yu B., Li S., Qiu W.Y., Wang M.H., Du J.W., Zhang Y. (2018). Prediction of subcellular location of apoptosis proteins by incorporating PsePSSM and DCCA coefficient based on LFDA dimensionality reduction. BMC Genomics.

[19] He H., Garcia E.A. (2009). Learning from imbalanced data. IEEE T Knowl Data En.

[20] Li W., Jaroszewski L., Godzik A. (2001). Clustering of highly homologous sequences to reduce the size of large protein databases. Bioinformatics.

[21] Zhou YZ, Gao Y, Zheng YY. Prediction of protein-protein interactions using local description of amino acid sequence. In: Zhou M., Tan H. (eds) Advances in Computer Science and Education Applications. Communications in Computer and Information Science, vol 202. Springer, Berlin, Heidelberg; 2011, p.254–62.

[22] Fan R.E., Chang K.W., Hsieh C.J., Wang X.R., Lin C.J. (2008). LIBLINEAR: a library for large linear classification. J Mach Learn Res.

[23] Friedman J.H. (2001). Greedy function approximation: a gradient boosting machine. Ann Stat.

[24] Friedman J.H. (2002). Stochastic gradient boosting. Comput Stat Data An.

[25] Pedregosa F., Varoquaux G., Gramfort A., Michel V., Thirion B., Grisel O. (2011). Scikit-learn: machine learning in python. J Mach Learn Res.

[26] Wang X., Yu B., Ma A., Chen C., Liu B., Ma Q. (2019). Protein-protein interaction sites prediction by ensemble random forests with synthetic minority oversampling technique. Bioinformatics.

[27] Davis J, Goadrich M. The relationship between Precision-Recall and ROC curves. In Proceedings of the 23rd International Conference on Machine Learning; 2006.

[28] Zhang D, Zhou ZH, Chen S. Semi-supervised dimensionality reduction. SIAM International Conference on Data Mining; 2007.

[29] Wold S., Esbensen K., Geladi P. (1987). Principal component analysis. Chemomet Intell Lab Syst.

[30] Schölkopf B., Smola A., Müller K.R. (1998). Nonlinear component analysis as a kernel eigenvalue problem. Neural Comput.

[31] Pournara I., Wernisch L. (2007). Factor analysis for gene regulatory networks and transcription factor activity profiles. BMC Bioinformatics.

[32] Peng H., Long F., Ding C. (2005). Feature selection based on mutual information: criteria of max-dependency, max-relevance, and min-redundancy. IEEE Trans Pattern Anal Mach Intell.

[33] Fleuret F. (2004). Binary feature selection with conditional mutual information. J Mach Learn Res.

[34] Nigsch F., Bender A., van Buuren B., Tissen J., Nigsch E., Mitchell J.B.O. (2006). Melting point prediction employing k-nearest neighbor algorithms and genetic parameter optimization. J Chem Inf Model.

[35] Friedman N., Geiger D., Pazzanzi M. (1997). Bayesian network classifiers. Mach Learn.

[36] Vapnik V.N. (1995). The nature of statistical learning theory.

[37] Breiman L. (2001). Random forest. Mach Learn.

[38] Yang L., Xia J.F., Gui J. (2010). Prediction of protein-protein interactions from protein sequence using local descriptors. Protein Pept Lett.

[39] You Z.H., Chan K.C.C., Hu P. (2015). Predicting protein-protein interactions from primary protein sequences using a novel multi-scale local feature representation scheme and the random forest. PLoS One.

[40] Wong L, You ZH, Li S, Huang YA, Liu G. Detection of protein-protein interactions from amino acid sequences using a rotation forest model with a novel PR-LPQ descriptor. International Conference on Intelligent Computing; Springer, Cham, 2015.

[41] Ding Y., Tang J., Guo F. (2016). Predicting protein-protein interactions via multivariate mutual information of protein sequences. BMC Bioinformatics.

[42] You Z.H., Li X., Chan K.C.C. (2017). An improved sequence-based prediction protocol for protein-protein interactions using amino acids substitution matrix and rotation forest ensemble classifiers. Neurocomputing.

[43] Nanni L. (2005). Fusion of classifiers for predicting protein-protein interactions. Neurocomputing.

[44] Nanni L., Lumini A. (2006). An ensemble of K-local hyperplanes for predicting protein-protein interactions. Bioinformatics.

[45] Huang Y.A., You Z.H., Gao X., Wong L., Wang L. (2015). Using weighted sparse representation model combined with discrete cosine transformation to predict protein-protein interactions from protein sequence. Biomed Res Int.

[46] Yang X.H., Kovalenko O.V., Kolesnikova T.V., Andzelm M.M., Rubinstein E., Strominger J.L. (2006). Contrasting effects of EWI proteins, integrins, and protein palmitoylation on cell surface CD9 organization. J Biol Chem.

[47] Stelzl U., Worm U., Lalowski M., Haenig C., Brembeck F.H., Goehler H. (2005). A human protein-protein interaction network: a resource for annotating the proteome. Cell.

[48] Shen J., Zhang J., Luo X., Zhu W., Yu K., Chen K. (2007). Predicting protein-protein interactions based only on sequences information. Proc Natl Acad Sci U S A.

[49] Charrin S., Manié S., Oualid M., Billard M., Boucheix C., Rubinsteina E. (2002). Differential stability of tetraspanin/tetraspanin interactions: role of palmitoylation. FEBS Lett.

[50] Bai J., Brody J.R., Kadkol S.S., Pasternack G.R. (2001). Tumor suppression and potentiation by manipulation of pp32 expression. Oncogene.

[51] Luo W., Lin S.C. (2004). Axin: a master scaffold for multiple signaling pathways. Neurosignals.

