# Supplementary material for: GTB-PPI: Predict Protein–protein Interactions Based on L1-regularized Logistic Regression and Gradient Tree Boosting
Source: Genomics Proteomics Bioinformatics. 2021 Jan 27;18(5):582–92. doi: 10.1016/j.gpb.2021.01.001 (PMC8377384; doi:10.1016/j.gpb.2021.01.001)
Supplement: Supplementary File S1 — Feature extraction methods. [file mmc1.docx]

**File S1 Feature extraction methods**

**Pseudo amino acid composition**

To extract the frequency and position information, Chou et al. [1] proposed a Pseudo amino acid composition (PseAAC) method. The feature vector of PseAAC model can be expressed as:

$X=[x_{1},x_{2},\cdot\cdot\cdot,x_{19},x_{20},x_{20+1},\cdot\cdot\cdot,x_{20+\lambda}]^{T} (\lambda<L)$ (1)

where $L$ indicates the sequence length.

$x_{u}=\left\{\begin{matrix} \frac{f_{u}}{\sum_{i=1}^{20} f_{i}+\omega\sum_{j=1}^{\lambda} \theta_{j}}, (1\leq u\leq20) \\ \frac{\omega\theta_{u-20}}{\sum_{i=1}^{20} f_{i}+\omega\sum_{j=1}^{\lambda} \theta_{j}}, (20+1\leq u\leq20+\lambda) \end{matrix}\right.$ (2)

where $f_{u}$ is the normalized frequency of $u\text{-}th$ amino acid and $\theta_{j}$ is the $j\text{-}th$ sequence correlation factor. $\omega$ is set to 0.05 [1].

**Pseudo position-specific scoring matrix**

Evolutionary information embedded in the PSSM has been applied in subcellular location prediction [2]. We used the PSI-BLAST tool [3] to search the non-redundant database Swiss-Prot to obtain PSSM of the protein sequence. During the running process, the PSI-BLAST parameter e-value threshold and the maximum number of iterations is set as 0.001 and 3, respectively.

First, the elements of the PSSM matrix are transformed into the [0, 1] interval via $f(x)$ sigmoid function.

$f(x)=\frac{1}{1+e^{-x}}$ (3)

To make PSSM become a size-uniform vector. The protein sample can be expressed

$\bar{P}_{PSSM}=\bar{p}_{1}\bar{p}_{2}\cdot\cdot\cdot\bar{p}_{20}$ (4)

where $\bar{p}_{j}=\frac{1}{L}\sum_{i=1}^{L} p_{i,j}$ represents the average score of the $j$ amino acid. However, only using $\bar{P}_{PSSM}$ could discard order information of PSSM. According to the pseudo amino acid composition, which was proposed by Shen and Chou [4], we obtain the Pseudo position-specific scoring matrix (PsePSSM) feature vector by Equation (5).

$\bar{p}_{j}=\left\{\begin{matrix} \frac{1}{L}\sum_{i=1}^{L} a_{i,j} j=1,2,\cdot\cdot\cdot,20, \zeta=0 \\ \frac{1}{L-\zeta}\sum_{i=1}^{L-\zeta} (a_{i,j}-a_{i+\zeta,j})^{2} j=1,2,\cdot\cdot\cdot,20, \zeta<L \end{matrix}\right.$ (5)

From Equation (5), each protein sequence can generate $20\text{+}20\times\xi$the dimensional feature vector.

**Reduced sequence and index-vectors**

The reduced alphabet schemes can effectively provide valuable information to represent PPIs. Xu et al. [5] extracted the amino acid sequence feature information using reduced sequence and index-vectors (RSIV) combined with q-FP and CMV feature extraction methods to predict therapeutic peptides. The amino acid sequence group coding in RSIV is shown in Table 1.

**Table 1 Amino acid classification according to different physicochemical property**

| **Property** | **Classifications** |
| --- | --- |
| Polarity/acidity | DE/ RHK/ WYF/ SCMNQT/ GAVLIP |
| Acidity | DE/ KHR/ ACFGILMNPQSTVWY |
| Secondary structure | EHALMQKR/ VTIYCWF/ GDNPS |
| Charge | KR/ AVNCQGHILMFPSTWY/ DE |
| DHP | PALVIFWM/ QSTYCNG/ HKR/ DE |
| Hydrophobicity | RKEDQN/ GASTPHY/ CLVIMFW |

For instance, 20 amino acid residues are classified into four groups according to DHP. A represents {P, A, L, V, I, F, W, M}; B represents {Q, S, T, Y, C, N, G}, C represents {H, K, R}, D represents {D, E}. For protein {MPNDNKTPNRSSTPKFTKKPVTPNDKIPEREEKSN}. The reduced sequence is {AABDBCBABCBBBACABCCAABABDCAADCDDCBB}. In the reduced sequence, the frequency of 'A' is 10; the 'B' is 12, the 'C' is 8, and the 'D' is 5. The frequency of di-character 'AA' is 3; the frequency 'AB' is 5; …; the frequency 'AD' is 1. And in this case, the frequency of 'DD' is 1.

Thus, RSIV can generate two types of index-vectors $V_{1}$ and $V_{2}$, which are shown in Equation (6) and (7).

$V_{1}=(\frac{|a_{11}|}{|R_{1}|},\frac{|a_{12}|}{|R_{1}|},\cdot\cdot\cdot,\frac{|a_{mk}|}{|R_{m}|})$ (6)

where $m$ indicates the number of groups whose value is 4 according to DHP. $|R_{i}|$ indicates the frequency of $R_{i}$. $\text{|}a_{mk}|$ indicates the frequency of $k\text{-}th$ character in the $m\text{-}th$ kind of group of the reduced sequence. The dimension of the vector $V_{1}$ is $20\times6\text{=}120$.

The encoding process of the vector $V_{2}$ is shown as

$V_{2}=(\frac{|R_{1}R_{1}|}{|R_{1}|},\frac{|R_{1}R_{2}|}{|R_{1}|},\cdot\cdot\cdot,\frac{|R_{m}R_{j}|}{|R_{m}|})$ (7)

**Autocorrelation descriptors**

In GTB-PPI model, three autocorrelation descriptors (AD) are selected: Morean-Broto autocorrelation (MBA), Moran autocorrelation (MA), Geary autocorrelation (GA) [6].

$NMBA(l)=\frac{MBA(l)}{N-l}, l=1,2,\cdot\cdot\cdot,lag$ (8)

where $MBA(l)=\sum_{i=1}^{N-l} P(AA_{i})P(AA_{i+l})$. $P(AA_{i})$ and $P(AA_{i+l})$ are standardized property values at the position of amino acid $AA_{i}$ and $AA_{i+l}$, respectively. The $lag$ is the parameter needs to be adjusted.

$MA(l)=\frac{\frac{1}{N-l}\sum_{i=1}^{N-l} (P(AA_{i})-\mu)(P(AA_{i\text{+}l})-\mu)}{\frac{1}{N}\sum_{i=1}^{N} (P(AA_{i})-\mu)^{2}}, l=1,2,\cdot\cdot\cdot,lag$ (9)

where $\mu$ is the mean value of each amino acid.

$GA(l)=\frac{\frac{1}{2\text{（}N-l)}\sum_{i=1}^{N-l} (P(AA_{i})-P(AA_{i+l}))^{2}}{\frac{1}{N}\sum_{i=1}^{N} (P(AA_{i})-\mu)^{2}}, l=1,2,\cdot\cdot\cdot,lag$ (10)

**References**

[1] Chou KC. Prediction of protein cellular attributes using pseudo-amino acid composition. Proteins 2001;43:246–55.

[2] Yu B, Shan L, Qiu WY, Wang MH, Du JW, Zhang Y, et al. Prediction of subcellular location of apoptosis proteins by incorporating PsePSSM and DCCA coefficient based on LFDA dimensionality reduction. BMC Genomics 2018;19:478.

[3] Altschul SF, Madden TL, Schäffer AA, Zhang JH, Zhang Z, Miller W, et al. Gapped BLAST and PSI-BLAST: a new generation of protein database search programs. Nucleic Acids Res 1997;25:3389–402.

[4] Shen HB, Chou KC. Nuc-PLoc: a new web-server for predicting protein subnuclear localization by fusing PseAA composition and PsePSSM. Protein Eng Des Sel 2007;20:561.

[5] Xu C, Ge L, Zhang Y, Dehmer M, Gutman I. Prediction of peptides by incorporating q-Wiener index into Chou's general PseAAC. J Biomed Inform 2017;75:63–9.

[6] Chen Z, Zhao P, Li F, Leier A, Marquez-Lago TT, Wang Y, et al. iFeature: a python package and web server for features extraction and selection from protein and peptide sequences. Bioinformatics 2018;34:2499–502.
