# Supplementary material for: GTB-PPI: Predict Protein–protein Interactions Based on L1-regularized Logistic Regression and Gradient Tree Boosting
Source: Genomics Proteomics Bioinformatics. 2021 Jan 27;18(5):582–92. doi: 10.1016/j.gpb.2021.01.001 (PMC8377384; doi:10.1016/j.gpb.2021.01.001)
Supplement: Supplementary File S2 — Parameter selection of ξ and lag. [file mmc2.docx]

**File S2 Parameter selection of $\xi$ and $lag$**

For the selection of parameter $\xi$ in PsePSSM, $\xi$ values are different when two datasets achieve the highest overall prediction accuracy. The optimal $\xi$ value for the *H. pylori* dataset is 3, while the optimal $\xi$ value for the *S. cerevisiae* dataset is 9. The parameter $\xi$ is set as 9 in PsePSSM to extract the evolutionary information. For the selection of parameter $lag$ in AD, the optimal $\xi$ values for the *S. cerevisiae* and *H. pylori* datasets are the same, both of which are 11. Based on above discussion, in the GTB-PPI model, the $\lambda$ value of PseAAC is set to 11, the $\xi$ value of PsePSSM is set to 9, and the $lag$ value of AD is set to 11.
