# Supplementary material for: GTB-PPI: Predict Protein–protein Interactions Based on L1-regularized Logistic Regression and Gradient Tree Boosting
Source: Genomics Proteomics Bioinformatics. 2021 Jan 27;18(5):582–92. doi: 10.1016/j.gpb.2021.01.001 (PMC8377384; doi:10.1016/j.gpb.2021.01.001)
Supplement: Supplementary File S3 — Parameter optimization of L1-RLR. [file mmc3.docx]

**File S3 Parameter optimization of L1-RLR**

Although feature fusion can acquire important, valuable feature information in the process of PPI prediction, the increase of dimension inevitably generates some unimportant features for classification. According to Equation (1), different penalty parameter $C$ values can be selected to determine the different subset. The penalty $C$ is set as 0.1, 0.3, 0.5, 0.8, 1, 1.2, 1.5, and 2, respectively, and the GTB classifier is employed to predict PPIs via five-fold cross-validation. The number of original features and selected feature subsets of PseAAC, PsePSSM, RSIV, and AD are shown in Figure S1 and Figure S2. On the *S. cerevisiae* and *H. pylori* datasets, GTB-PPI achieve the best prediction performance $C\text{=}1$. When the $C$ value is 1, 331 optimal features are selected for *S. cerevisiae,* and 199 optimal features have been remained for *H. pylori*.
