# Supplementary material for: GTB-PPI: Predict Protein–protein Interactions Based on L1-regularized Logistic Regression and Gradient Tree Boosting
Source: Genomics Proteomics Bioinformatics. 2021 Jan 27;18(5):582–92. doi: 10.1016/j.gpb.2021.01.001 (PMC8377384; doi:10.1016/j.gpb.2021.01.001)
Supplement: Supplementary Table S1 [file mmc4.docx]

**Table S1 Number of unique proteins for each dataset**

| **Dataset** | **No. of protein pairs** | **No. of unique proteins** |
| --- | --- | --- |
| *S. cerevisiae* | 11,188 | 2530 |
| *H. pylori* | 2916 | 1428 |
| *C. elegans* | 4013 | 2629 |
| *E. coli* | 6954 | 1830 |
| *H. sapiens* | 1412 | 1083 |
| *M. musculus* | 313 | 355 |
