# Supplementary material for: GTB-PPI: Predict Protein–protein Interactions Based on L1-regularized Logistic Regression and Gradient Tree Boosting
Source: Genomics Proteomics Bioinformatics. 2021 Jan 27;18(5):582–92. doi: 10.1016/j.gpb.2021.01.001 (PMC8377384; doi:10.1016/j.gpb.2021.01.001)
Supplement: Supplementary Table S2 [file mmc5.docx]

**Table S2 Performance comparison with different $\lambda$ values on PPI datasets**

| **Dataset** | **Evaluation** | **$\lambda$** | | | | | |
| --- | --- | --- | --- | --- | --- | --- | --- |
|  |  | **1** | **3** | **5** | **7** | **9** | **11** |
| *S. cerevisiae* | ACC | 94.38 | 94.57 | 94.59 | 94.50 | **94.87** | 94.60 |
|  | Recall | 91.99 | 91.96 | 92.06 | 92.08 | 92.42 | 92.08 |
|  | Precision | 96.61 | 97.02 | 96.97 | 96.78 | 97.18 | 96.98 |
|  | MCC | 0.8886 | 0.8926 | 0.8930 | 0.8911 | 0.8985 | 0.8933 |
| *H. pylori* | ACC | 88.72 | 88.72 | 88.41 | 89.09 | 89.16 | **89.44** |
|  | Recall | 87.72 | 87.45 | 86.83 | 87.92 | 88.75 | 88.89 |
|  | Precision | 89.60 | 89.78 | 89.67 | 90.08 | 89.49 | 89.90 |
|  | MCC | 0.7753 | 0.7751 | 0.7686 | 0.7827 | 0.7833 | 0.7890 |

*Note*: The numbers in bold mean maximum. ACC, overall prediction accuracy; MCC, Matthews correlation coefficient.
