# Supplementary material for: GTB-PPI: Predict Protein–protein Interactions Based on L1-regularized Logistic Regression and Gradient Tree Boosting
Source: Genomics Proteomics Bioinformatics. 2021 Jan 27;18(5):582–92. doi: 10.1016/j.gpb.2021.01.001 (PMC8377384; doi:10.1016/j.gpb.2021.01.001)
Supplement: Supplementary Table S3 [file mmc6.docx]

**Table S3 Performance comparison with different $\xi$ values on PPI datasets**

| **Dataset** | **Evaluation** | **$\xi$** | | | | | |
| --- | --- | --- | --- | --- | --- | --- | --- |
|  |  | **1** | **3** | **5** | **7** | **9** | **11** |
| *S. cerevisiae* | ACC | 94.58 | 94.71 | 94.87 | 94.68 | **94.98** | 94.75 |
|  | Recall | 92.08 | 92.08 | 92.31 | 92.06 | 92.47 | 92.08 |
|  | Precision | 96.94 | 97.19 | 97.29 | 97.15 | 97.37 | 97.28 |
|  | MCC | 0.8928 | 0.8955 | 0.8986 | 0.8949 | 0.9009 | 0.8964 |
| *H. pylori* | ACC | 87.18 | **88.85** | 88.62 | 88.00 | 88.20 | 88.75 |
|  | Recall | 86.97 | 89.02 | 88.96 | 88.00 | 88.96 | 89.09 |
|  | Precision | 87.34 | 88.74 | 88.43 | 88.05 | 87.74 | 88.49 |
|  | MCC | 0.7436 | 0.7773 | 0.7729 | 0.7603 | 0.7648 | 0.7751 |

*Note*: The numbers in bold mean maximum. ACC, overall prediction accuracy; MCC, Matthews correlation coefficient.
