# Supplementary material for: GTB-PPI: Predict Protein–protein Interactions Based on L1-regularized Logistic Regression and Gradient Tree Boosting
Source: Genomics Proteomics Bioinformatics. 2021 Jan 27;18(5):582–92. doi: 10.1016/j.gpb.2021.01.001 (PMC8377384; doi:10.1016/j.gpb.2021.01.001)
Supplement: Supplementary Table S4 [file mmc7.docx]

**Table S4 Prediction results with different $lag$ values on PPI datasets**

| **Dataset** | **Evaluation** | **$lag$** | | | | | |
| --- | --- | --- | --- | --- | --- | --- | --- |
|  |  | **1** | **3** | **5** | **7** | **9** | **11** |
| *S. cerevisiae* | ACC | 93.73 | 94.12 | 94.52 | 94.37 | 94.32 | **94.53** |
|  | Recall | 91.37 | 91.20 | 91.72 | 91.33 | 91.29 | 91.47 |
|  | Precision | 95.91 | 96.85 | 97.16 | 97.25 | 97.18 | 97.43 |
|  | MCC | 0.8757 | 0.8839 | 0.8918 | 0.8890 | 0.8880 | 0.8923 |
| *H. pylori* | ACC | 85.60 | 88.30 | 89.03 | 89.44 | 89.10 | **89.68** |
|  | Recall | 84.43 | 86.70 | 88.20 | 88.75 | 88.13 | 89.44 |
|  | Precision | 86.50 | 89.58 | 89.68 | 90.01 | 89.95 | 89.92 |
|  | MCC | 0.7126 | 0.7667 | 0.7809 | 0.7890 | 0.7828 | 0.7938 |

*Note*: The numbers in bold mean maximum. ACC, overall prediction accuracy; MCC, Matthews correlation coefficient.
