# Supplementary material for: GTB-PPI: Predict Protein–protein Interactions Based on L1-regularized Logistic Regression and Gradient Tree Boosting
Source: Genomics Proteomics Bioinformatics. 2021 Jan 27;18(5):582–92. doi: 10.1016/j.gpb.2021.01.001 (PMC8377384; doi:10.1016/j.gpb.2021.01.001)
Supplement: Supplementary Table S5 [file mmc8.docx]

**Table S5 Performance of principle component analysis with different contribution rates**

| **Dataset** | **Evaluation** | **The rate of contribution (%)** | | | |
| --- | --- | --- | --- | --- | --- |
|  |  | **80** | **85** | **90** | **95** |
| *S. cerevisiae* | ACC | 86.16 | 86.59 | 86.91 | **87.62** |
|  | Recall | 85.63 | 85.88 | 86.04 | 86.29 |
|  | Precision | 86.56 | 87.12 | 87.58 | 88.65 |
|  | MCC | 0.7234 | 0.7320 | 0.7384 | 0.7527 |
| *H. pylori* | ACC | 79.42 | 79.80 | **80.18** | 79.25 |
|  | Recall | 77.09 | 78.67 | 77.91 | 76.48 |
|  | Precision | 80.90 | 80.53 | 81.60 | 80.98 |
|  | MCC | 0.5893 | 0.5964 | 0.6043 | 0.5861 |

*Note*: The numbers in bold mean maximum. ACC, overall prediction accuracy; MCC, Matthews correlation coefficient.
