# Supplementary material for: GTB-PPI: Predict Protein–protein Interactions Based on L1-regularized Logistic Regression and Gradient Tree Boosting
Source: Genomics Proteomics Bioinformatics. 2021 Jan 27;18(5):582–92. doi: 10.1016/j.gpb.2021.01.001 (PMC8377384; doi:10.1016/j.gpb.2021.01.001)
Supplement: Supplementary Table S6 [file mmc9.docx]

**Table S6 Performance of kernel principle component analysis with different** **contribution rates**

| **Dataset** | **Evaluation** | **The rate of contribution (%)** | | | |
| --- | --- | --- | --- | --- | --- |
|  |  | **80** | **85** | **90** | **95** |
| *S. cerevisiae* | ACC | 85.49 | 85.37 | 85.36 | **85.63** |
|  | Recall | 84.93 | 85.15 | 84.97 | 85.23 |
|  | Precision | 85.90 | 85.52 | 85.65 | 85.94 |
|  | MCC | 0.7100 | 0.7075 | 0.7073 | 0.7127 |
| *H. pylori* | ACC | 79.08 | 78.88 | **80.42** | 78.50 |
|  | Recall | 77.16 | 78.19 | 79.15 | 77.92 |
|  | Precision | 80.26 | 79.27 | 81.31 | 78.91 |
|  | MCC | 0.5823 | 0.5776 | 0.6092 | 0.5710 |

*Note*: The numbers in bold mean maximum. ACC, overall prediction accuracy; MCC, Matthews correlation coefficient.
