# Supplementary material for: GTB-PPI: Predict Protein–protein Interactions Based on L1-regularized Logistic Regression and Gradient Tree Boosting
Source: Genomics Proteomics Bioinformatics. 2021 Jan 27;18(5):582–92. doi: 10.1016/j.gpb.2021.01.001 (PMC8377384; doi:10.1016/j.gpb.2021.01.001)
Supplement: Supplementary Table S7 [file mmc10.docx]

**Table S7 Comparison of prediction results on different dimensional reduction methods**

| **Dataset** | **Evaluation** | **Method** | | | | | | | |
| --- | --- | --- | --- | --- | --- | --- | --- | --- | --- |
|  |  | **L1-RLR** | | **SSDR** | **PCA** | **KPCA** | **FA** | **mRMR** | **CMIM** |
| *S. cerevisiae* | ACC | 95.15 | 87.02 | | 87.62 | 85.63 | 88.94 | 92.55 | 92.32 |
|  | Recall | 92.21 | 85.47 | | 86.29 | 85.23 | 87.20 | 90.01 | 89.65 |
|  | Precision | 97.97 | 88.26 | | 88.65 | 85.94 | 90.36 | 94.84 | 94.74 |
|  | MCC | 0.9045 | 0.7410 | | 0.7527 | 0.7127 | 0.7794 | 0.8523 | 0.8479 |
| *H. pylori* | ACC | 90.47 | 85.25 | | 79.25 | 78.50 | 80.28 | 87.76 | 78.43 |
|  | Recall | 89.99 | 77.16 | | 76.47 | 77.92 | 77.02 | 87.59 | 78.33 |
|  | Precision | 91.15 | 92.12 | | 80.98 | 78.91 | 82.40 | 87.91 | 78.54 |
|  | MCC | 0.8100 | 0.7151 | | 0.5861 | 0.5710 | 0.6073 | 0.7553 | 0.5691 |

*Note*: ACC, overall prediction accuracy; MCC, Matthews correlation coefficient; L1-RLR, L1-regularized logistic regression; SSDR, semi-supervised dimension reduction; PCA, principal component analysis; KPCA, kernel principal component analysis; FA, factor analysis; mRMR, minimum redundancy maximum relevance; CMIM, conditional mutual information maximization.
