# Supplementary material for: GTB-PPI: Predict Protein–protein Interactions Based on L1-regularized Logistic Regression and Gradient Tree Boosting
Source: Genomics Proteomics Bioinformatics. 2021 Jan 27;18(5):582–92. doi: 10.1016/j.gpb.2021.01.001 (PMC8377384; doi:10.1016/j.gpb.2021.01.001)
Supplement: Supplementary Table S8 [file mmc11.docx]

**Table S8 AUROC and AUPRC on different dimensional reduction methods**

| **Dataset** | **Evaluation** | **L1-RLR** | **SSDR** | **PCA** | **KPCA** | **FA** | **mRMR** | **CMIM** |
| --- | --- | --- | --- | --- | --- | --- | --- | --- |
| *S. cerevisiae* | AUROC | 0.9875 | 0.9445 | 0.9420 | 0.9305 | 0.9568 | 0.9770 | 0.9769 |
|  | AUPRC | 0.9847 | 0.9388 | 0.9386 | 0.9271 | 0.9510 | 0.9728 | 0.9717 |
| *H. pylori* | AUROC | 0.9559 | 0.9238 | 0.8706 | 0.8803 | 0.8824 | 0.9461 | 0.8726 |
|  | AUPRC | 0.9498 | 0.9077 | 0.8485 | 0.8728 | 0.8665 | 0.9464 | 0.8516 |

*Note*: AUROC, area under receiver operating characteristic curve; AUPRC, area under precision-recall curve**;** L1-RLR, L1-regularized logistic regression; SSDR, semi-supervised dimension reduction; PCA, principal component analysis; KPCA, kernel principal component analysis; FA, factor analysis; mRMR, minimum redundancy maximum relevance; CMIM, conditional mutual information maximization.
