# Supplementary material for: GTB-PPI: Predict Protein–protein Interactions Based on L1-regularized Logistic Regression and Gradient Tree Boosting
Source: Genomics Proteomics Bioinformatics. 2021 Jan 27;18(5):582–92. doi: 10.1016/j.gpb.2021.01.001 (PMC8377384; doi:10.1016/j.gpb.2021.01.001)
Supplement: Supplementary Table S9 [file mmc12.docx]

**Table S9 Performance of *K* nearest neighbors with different size of neighbors**

| **Dataset** | **Evaluation** | **The size of neighbors** | | | | | | |
| --- | --- | --- | --- | --- | --- | --- | --- | --- |
|  |  | **1** | **3** | **5** | **7** | **9** | **15** | **20** |
| *S. cerevisiae* | ACC | 82.40 | 83.74 | **83.82** | 83.39 | 83.17 | 81.89 | 81.43 |
|  | Recall | 81.09 | 81.69 | 81.98 | 82.17 | 82.77 | 83.57 | 83.68 |
|  | Precision | 83.29 | 85.18 | 85.13 | 84.24 | 83.46 | 80.86 | 80.10 |
|  | MCC | 0.6484 | 0.6754 | 0.6770 | 0.6682 | 0.6638 | 0.6384 | 0.6295 |
| *H. pylori* | ACC | **74.63** | 73.77 | 73.97 | 72.43 | 71.33 | 71.06 | 70.96 |
|  | Recall | 88.55 | 91.02 | 91.15 | 90.95 | 90.60 | 91.02 | 91.15 |
|  | Precision | 69.28 | 67.69 | 67.86 | 66.41 | 65.42 | 65.16 | 64.99 |
|  | MCC | 0.5129 | 0.5064 | 0.5106 | 0.4831 | 0.4625 | 0.4594 | 0.4580 |

*Note*: The numbers in bold mean maximum. ACC, overall prediction accuracy; MCC, Matthews correlation coefficient.
