# Supplementary material for: GTB-PPI: Predict Protein–protein Interactions Based on L1-regularized Logistic Regression and Gradient Tree Boosting
Source: Genomics Proteomics Bioinformatics. 2021 Jan 27;18(5):582–92. doi: 10.1016/j.gpb.2021.01.001 (PMC8377384; doi:10.1016/j.gpb.2021.01.001)
Supplement: Supplementary Table S10 [file mmc13.docx]

**Table S10 Performance of random forest with different size of base decision trees**

| **Dataset** | **Evaluation** | **The size of base decision trees** | | | | |
| --- | --- | --- | --- | --- | --- | --- |
|  |  | **50** | **100** | **500** | **1000** | **2000** |
| *S. cerevisiae* | ACC | 92.69 | 92.70 | 92.78 | 92.79 | **92.84** |
|  | Recall | 88.24 | 88.38 | 88.34 | 88.29 | 88.38 |
|  | Precision | 96.86 | 96.74 | 96.94 | 97.04 | 97.04 |
|  | MCC | 0.8572 | 0.8572 | 0.8590 | 0.8594 | 0.8603 |
| *H. pylori* | ACC | 87.76 | 88.72 | **89.06** | 88.75 | 88.72 |
|  | Recall | 88.41 | 89.78 | 89.64 | 89.71 | 89.44 |
|  | Precision | 87.30 | 87.96 | 88.67 | 88.06 | 88.21 |
|  | MCC | 0.7554 | 0.7749 | 0.7818 | 0.7754 | 0.7748 |

*Note*: The numbers in bold mean maximum. ACC, overall prediction accuracy; MCC, Matthews correlation coefficient.
