# Supplementary material for: GTB-PPI: Predict Protein–protein Interactions Based on L1-regularized Logistic Regression and Gradient Tree Boosting
Source: Genomics Proteomics Bioinformatics. 2021 Jan 27;18(5):582–92. doi: 10.1016/j.gpb.2021.01.001 (PMC8377384; doi:10.1016/j.gpb.2021.01.001)
Supplement: Supplementary Table S11 [file mmc14.docx]

**Table S11** **Prediction results of different classifiers on S. *cerevisiae* and *H. pylori* datasets**

| **Dataset** | **Model** | **ACC (%)** | **Recall (%)** | **Precision (%)** | **MCC** |
| --- | --- | --- | --- | --- | --- |
| *S. cerevisiae* | KNN | 83.82 ± 0.64 | 81.98 ± 0.80 | 85.13 ± 1.04 | 0.6770 ± 0.0131 |
|  | NB | 70.86 ± 1.81 | 62.53 ± 3.18 | 75.01 ± 1.60 | 0.4233 ± 0.0351 |
|  | SVM | 89.94 ± 0.44 | 88.93 ± 0.65 | 90.77 ± 0.59 | 0.7991 ± 0.0088 |
|  | RF | 92.78 ± 0.48 | 88.34 ± 0.61 | 96.94 ± 0.64 | 0.8590 ± 0.0096 |
|  | GTB | 95.15 ± 0.25 | 92.21 ± 0.36 | 97.97 ± 0.60 | 0.9045 ± 0.0053 |
| *H. pylori* | KNN | 73.97 ± 1.87 | 91.15 ± 1.65 | 67.86 ± 1.61 | 0.5106 ± 0.0372 |
|  | NB | 69.34 ± 1.42 | 82.58 ± 0.79 | 65.32 ± 1.47 | 0.4012 ± 0.0267 |
|  | SVM | 84.54 ± 1.53 | 83.88 ± 2.08 | 85.05 ± 2.22 | 0.6912 ± 0.0311 |
|  | RF | 89.06 ± 0.81 | 89.64 ± 1.77 | 88.67 ± 1.72 | 0.7818 ± 0.0161 |
|  | GTB | 90.47 ± 0.84 | 91.15 ± 1.42 | 89.99 ± 2.06 | 0.8100 ± 0.0163 |

*Note*: ACC, overall prediction accuracy; MCC, Matthews correlation coefficient; GTB, gradient tree boosting; KNN, *K* nearest neighbors; NB, Naïve Bayes; SVM, support vector machine; RF, random forest.
