# Supplementary material for: GTB-PPI: Predict Protein–protein Interactions Based on L1-regularized Logistic Regression and Gradient Tree Boosting
Source: Genomics Proteomics Bioinformatics. 2021 Jan 27;18(5):582–92. doi: 10.1016/j.gpb.2021.01.001 (PMC8377384; doi:10.1016/j.gpb.2021.01.001)
Supplement: Supplementary Table S12 [file mmc15.docx]

**Table S12 AUROC and AUPRC of different classifiers on S. *cerevisiae* and *H. pylori* datasets**

| **Dataset** | **Evaluation** | **Method** | | | | |
| --- | --- | --- | --- | --- | --- | --- |
|  |  | **KNN** | **NB** | **SVM** | **RF** | **GTB** |
| *S. cerevisiae* | AUROC | 0.9172 | 0.7922 | 0.9618 | 0.9762 | 0.9875 |
|  | AUPRC | 0.9006 | 0.7921 | 0.9581 | 0.9709 | 0.9847 |
| *H. pylori* | AUROC | 0.8683 | 0.7775 | 0.9214 | 0.9509 | 0.9559 |
|  | AUPRC | 0.8531 | 0.7625 | 0.9182 | 0.9477 | 0.9498 |

*Note*: GTB, gradient tree boosting; KNN, $K$ nearest neighbors; NB, Naïve Bayes; SVM, support vector machine; RF, random forest; AUROC, area under receiver operating characteristic curve; AUPRC, area under precision-recall curve.
